# BK Polyomavirus Subtypes IVc-1 and Ib-1 in Vietnamese Renal Transplant Recipients

**DOI:** 10.1128/MRA.00427-21

**Published:** 2021-08-26

**Authors:** Hang Thi Thu Dinh, Son Anh Ho, Toan Quoc Pham, Su Xuan Hoang

**Affiliations:** a Department of Microbiology and Pathogens, Institute of Biomedicine and Pharmacy, Vietnam Military Medical University, Hanoi, Vietnam; b Department of Nephrology, Military Hospital 103, Vietnam Military Medical University, Hanoi, Vietnam; KU Leuven

## Abstract

We report here the nearly complete genome sequences of two human BK polyomavirus (BKV) strains recovered from two Vietnamese renal allograft recipients and belonging to subtypes IVc1 (strain VN_PBK185) and Ib1 (strain VN_PBK212). The genome sequences of VN_PBK185 and VN_PBK212 were highly similar (99.9% nucleotide identity) to the reference BKV strains VNM-1 and VNM-9, respectively.

## ANNOUNCEMENT

Human BK polyomavirus (BKV) is a ubiquitous pathogen with worldwide distribution among humans. In the majority of cases, this virus does not cause any symptoms but only becomes an emerging opportunistic infectious pathogen in immunocompromised patients, especially individuals with kidney transplantation (1). Taxonomically, BKV belongs to the family *Polyomaviridae*, genus *Betapolyomavirus*, and has a non-enveloped capsid. The genome consists of a circular double-stranded DNA molecule about 5.1 kb long. Based on either genotyping or serotyping methods, BKV is classified into four genotypes (I to IV), with genotype I predominant worldwide, followed by genotype IV. Meanwhile, genotypes II and III are found at lower rates (2, 3). Phylogenetically, genotype I is further divided into four subtypes (Ia, Ib1, Ib2, and Ic), whereas genotype IV comprises six subtypes (IVa1, IVa2, IVb1, IVb2, IVc1, and IVc2) (4). Chen et al. identified the BKV prevalence rate as 24% in the urine of Vietnamese adults without immunosuppression, including genotypes I and IV (5). Here, nearly complete genome sequences of BKV were derived from the urine specimens of two renal transplant recipients in northern Vietnam.

Urine and blood samples were collected from two renal allograft recipients at Military Hospital 103, Vietnam Military Medical University, Hanoi, Vietnam, in 2019 (male patients aged 34 and 35 years old). These clinical samples were sent to our laboratory to measure the BKV DNA loads and for subsequent BKV genotyping. The purpose of the study was explained to each patient, and informed consent was obtained, after the study protocol was approved by the local ethics committee. Briefly, the BKV DNA loads in plasma and urine were >10^4^ copies/ml and 10^9^ copies/ml (patient 1) and >10^3^ copies/ml and 10^8^ copies/ml (patient 2), respectively, as quantified using the RealStar BKV PCR 1.0 kit (Altona Diagnostics, Germany). Next, DNA was extracted from the urine specimens, and a PCR amplicon of 5.1 kb was generated using the following forward and reverse primers: wBK_aF (672 to 700), 5′GAG GCT GCT GCT GCC ACA GGA TTT TCA GT 3′, and wBK_aR (680 to 651), 5′ AGC AGC CTC AGA TAC ACT GGC AAC TAG GTC 3′. The reaction mixture (50 μl) contained 5 μl DNA template, 1× Phusion master mix with high-fidelity (HF) buffer (Thermo Scientific, USA), 0.5 μM primer wBK_aF/R, and deionized water. The PCR conditions were 98°C for 2 min, followed by 35 cycles of 98°C for 20 s, 58°C for 20 s, and 72°C for 4 min, with a final extension at 72°C for 4 min. The PCR products were gel extracted (GeneJET gel extraction kit; Thermo Scientific, USA), purified, and subsequently used for direct Sanger sequencing with 12 primers (Table 1) using the Applied Biosystems Prism BigDye Terminator v1.1 kit.

**TABLE 1:** Primers used for this study

No.	Primer	Sequence (5′–3′)	Target gene	Nucleotide position^*c*^	Reference
1	wBK_aF^*a*^	GAGGCTGCTGCTGCCACAGGATTTTCAGT		672–700	This study
2	wBK_aR^*a*^	AGCAGCCTCAGATACACTGGCAACTAGGTC		680–651	This study
3	BKFuBa-IF^*b*^	GGGGGATCCAGATGAAAACCTTAGGGGCT		1731–59	6
4	BKFuBa-IR^*b*^	GGATCCCCCATTTCTGGGTTTAGGAAGCAT		1739–10	6
5	LT-BK-F^*b*^	CCAGCCTTTCCTTCCATTC	Large T	2994–3012	7
6	LT-BK-R^*b*^	CACTGTTTGCTACCTAAAATG	Large T	3621–3601	7
7	NC-BK-F^*b*^	ATTTCCCCAAATAGTTTTGCTAGGC	NC	28–52	8
8	NC-BK-R^*b*^	ACAGCAGGTAAAGCAGTGGT	NC	563–544	9
9	sTAg-F^*b*^	GCATTTCTTCYCTGGTCATAT	Large T and small T	3723–3743	This study
10	sTAg-R^*b*^	GTAGTAAGGGTGTGGAGGCTT	Large T and small T	81–61	This study
11	BK_S^*b*^	ATCAAAGAACTGCTCCTCAAT	VP1	1480–1501	10
12	BK_AS^*b*^	GCACTCCCTGCATTTCCAAGGG	VP1	2059–2038	10

a Primer used for genome amplification and sequencing.

b Primer used for genome sequencing.

c Nucleotide positions referenced according to the Dunlop strain, GenBank accession number V01108.1 or NC_001538.

**FIG 1:**
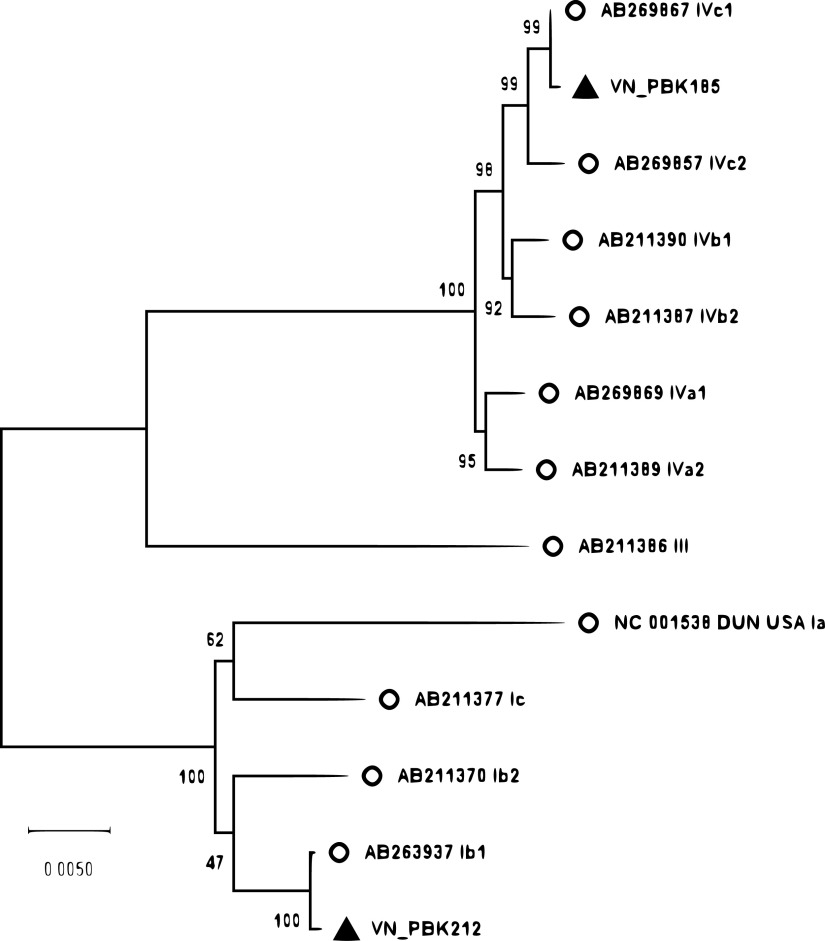
Maximum likelihood tree (Tamura-Nei model) of the MEGA X software based on the complete genome sequences of the 2 strains obtained in this study and 11 reference strains, including the BKV Dunlop sequence (GenBank accession number V01108.1 or NC_001538). The reliability of the tree topology was estimated by using 1,000 bootstrap replicates. The scale bar represents nucleotide substitutions per site. The triangles indicate the strains from this study. The circles indicate the complete sequences of the reference BKV strains.

All sequencing results were validated using BioEdit software, and the obtained sequences were assembled using Geneious v10.2 software. Mapping to the Dunlop BKV genome (GenBank accession number NC_001538), a contiguous sequence represented by 5,145 nucleotides (A:C:G:T = 1,543:1,014:1,015:1,573) contained a total GC content of 39.44% (patient 1), and a contiguous sequence represented by 5,139 nucleotides (A:C:G:T = 1,547:1,019:1,004:1,569) contained a total GC content of 39.37% (patient 2). Phylogenetic analysis showed that these BKV sequences belong to subtypes IVc1 (patient 1) and Ib1 (patient 2), with both sequences having 99.9% nucleotide sequence similarity to the reference strains VNM-1 and VNM-9 (AB269867 and AB263937, respectively) (Fig. 1). In conclusion, this study presented the nearly complete genome sequences of two BKV strains obtained from Vietnamese patients with renal transplantation using a simple, rapid and reliable protocol, which supported a complete genetic analysis of BKV.

### Data availability.

The complete genome sequences of BKV strains VN_PBK185 and VN_PBK212 have been deposited in the GenBank database under the accession numbers MW678842 and MW678843, respectively.
